# Patient-derived tissue slice grafts accurately depict response of high-risk primary prostate cancer to androgen deprivation therapy

**DOI:** 10.1186/1479-5876-11-199

**Published:** 2013-08-28

**Authors:** Hongjuan Zhao, Alan Thong, Rosalie Nolley, Stephen W Reese, Jennifer Santos, Alexandre Ingels, Donna M Peehl

**Affiliations:** 1 Department of Urology, Stanford University School of Medicine, Stanford, California, USA

**Keywords:** Prostate cancer, Androgen deprivation therapy, Tumorgrafts

## Abstract

**Background:**

Effective eradication of high-risk primary prostate cancer (HRPCa) could significantly decrease mortality from prostate cancer. However, the discovery of curative therapies for HRPCa is hampered by the lack of authentic preclinical models.

**Methods:**

We improved upon tumorgraft models that have been shown to predict drug response in other cancer types by implanting thin, precision-cut slices of HRPCa under the renal capsule of immunodeficient mice. Tissue slice grafts (TSGs) from 6 cases of HRPCa were established in mice. Following androgen deprivation by castration, TSGs were recovered and the presence and phenotype of cancer cells were evaluated.

**Results:**

High-grade cancer in TSGs generated from HRPCa displayed characteristic Gleason patterns and biomarker expression. Response to androgen deprivation therapy (ADT) was as in humans, with some cases exhibiting complete pathologic regression and others showing resistance to castration. As in humans, ADT decreased cell proliferation and prostate-specific antigen expression in TSGs. Adverse pathological features of parent HRPCa were associated with lack of regression of cancer in corresponding TSGs after ADT. Castration-resistant cancer cells remaining in TSGs showed upregulated expression of androgen receptor target genes, as occurs in castration-resistant prostate cancer (CRPC) in humans. Finally, a rare subset of castration-resistant cancer cells in TSGs underwent epithelial-mesenchymal transition, a process also observed in CRPC in humans.

**Conclusions:**

Our study demonstrates the feasibility of generating TSGs from multiple patients and of generating a relatively large number of TSGs from the same HRPCa specimen with similar cell composition and histology among control and experimental samples in an *in vivo* setting. The authentic response of TSGs to ADT, which has been extensively characterized in humans, suggests that TSGs can serve as a surrogate model for clinical trials to achieve rapid and less expensive screening of therapeutics for HRPCa and primary CRPC.

## Background

Mortality from prostate cancer (PCa) is confined to those men who have either advanced disease (distant metastases at initial presentation) or high-risk localized PCa (HRPCa) [1,2]. The definition of HRPCa is either a Gleason score of 8–10, pre-treatment serum prostate-specific antigen (PSA) > 20 ng/ml, or clinical stage of T3/T4 at diagnosis [3]. In addition, patients with at least two of the following criteria - a Gleason score of 7, pre-treatment serum PSA > 10 ng/ml, and a clinical stage of T2b/c - may also be considered high-risk [3]. Since < 5% of patients with newly diagnosed PCa have advanced metastatic disease, HRPCa, which comprises 15-40% of the overall PCa patient population, has become an important focus of novel therapeutic development [4,5].

Androgen receptor (AR) signaling plays a central role in all stages of PCa. For HRPCa, the standard of care is either radical prostatectomy or radiation therapy combined with androgen-deprivation therapy (ADT) [5-8]. In spite of treatment, up to 50% of these high-risk patients will inevitably progress to castration-resistant prostate cancer (CRPC), which is incurable, within 10 years [6,9-11]. One of the key mechanisms of resistance to ADT is the continued expression of AR by most CRPC and dependence on AR for growth [7-9,12]. Moreover, ADT induces epithelial-mesenchymal transition (EMT), a process that has been associated with aggressive clinical behavior in human PCa [13,14].

New primary therapies that can be used to eradicate HRPCa alone or in combination with ADT before CRPC arises, or to treat primary CRPC effectively after ADT, are urgently needed. Because experimental models of primary human PCa are extremely limited, new generations of compounds targeting AR signaling at different levels as well as other essential pathways in PCa have been developed using pre-clinical models of metastatic CRPC [15-18]. Whether these agents will be effective against primary HRPCa and derivative CRPC is not known. A realistic and representative *in vivo* model of HRPCa and primary CRPC is critical for pre-clinical assessment and comparison of different treatment options. Such a model will not only accelerate the discovery of effective therapies by minimizing the number of costly and time-consuming clinical trials, but also help enhance our understanding of mechanisms of therapeutic resistance.

Remarkable correlations between drug activity in “tumorgrafts” derived directly from patient tissues and clinical outcomes have been observed [19,20]. For instance, Hidalgo et al. demonstrated a notable correlation between drug activity in patient-derived tumorgrafts and clinical outcome in 14 types of advanced cancers [20]. Multiple groups have reported the ability to establish PCa tumorgrafts in mice under the skin or renal capsule, often through the use of minced pieces of tissue [21-25]. When minced fragments of tissue are used to generate grafts, it is impossible to know the composition of any given fragment (or even whether it contains cancer), due to the heterogeneous nature of prostate tissue. This, in turn, makes it impossible to ensure that tissues with similar composition are used in control and experimental groups, which, in turn, confounds interpretation of results. In addition, it is difficult to generate enough grafts from a single prostatectomy specimen to carry out experiments to test drugs with sufficient statistical power. Unfortunately for PCa research, metastatic tissue is also very difficult to obtain, and access to such tissue is predominantly limited to the few academic programs that support “rapid autopsy” programs. For all of the reasons stated above and more, tumorgrafts of PCa are not often included in studies such as that described above by Hidalgo et al. with multiple types of cancers (but no PCa) [20].

We developed methodology to establish tumorgrafts from thin, precision-cut tissue slices of human PCa to overcome at least some of the problems [26]. This novel *in vivo* tissue slice graft (TSG) model: 1) retains PCa histopathology, allowing for analysis of almost all of the cell types present in PCa and their interactions; 2) provides accurate assessment of the effects of interventions when tissues from the same specimen with similar cell composition are used as control and experimental samples; 3) ensures sufficient samples obtained for large experiments; and 4) permits optimal exchange of nutrients, oxygen, and drugs between TSG and the host.

Here we characterized TSGs generated from 6 HRPCa cases as well as the castration-resistant cancer that remained in TSGs from 3 of 5 cases after ADT. We focused on high-grade components of the tumors as the likely cause of recurrence and/or castration-resistance after primary therapy. The main questions we addressed were whether cancers in TSGs maintained in intact mice retained the histology and biomarker expression of parent tumors, and whether androgen deprivation affected cell proliferation, AR-regulated gene expression and EMT of cancers in TSGs similarly to that in humans. We provide evidence that TSGs are the first realistic model of primary HRPCa and CRPC that can be used with high predictive power to evaluate an exponentially growing number of molecularly targeted therapies and to discriminate the most effective therapeutics for further clinical development.

## Methods

### Ethics statement

All animal studies were approved by the Stanford Administrative Panel on Laboratory Animal Care (APLAC) and done in compliance with the regulations for animal studies at Stanford University. Patient-derived tissues were obtained immediately after surgery under a protocol approved by the Stanford Institutional Review Board. The participants provided their written informed consent to participate in this study.

### Patient samples

Clinical and histopathologic parameters of the donors are summarized in Table 1. None of the patients had chemical, hormonal, or radiation therapy prior to radical prostatectomy.

**Table 1:** Clinical and histopathologic variables of tissue donors

**ID**	**Pre-op PSA ng/ml**	**Gleason score**		**Clinical stage**	**Pathological stage**	**SV**^**1**^	**SM**^**2**^	**ECE**^**3**^
		**Biopsy**	**Prostatectomy**					
HRPCa-1	38.48	4+3	4+3	cT1c	pT3bN0	+	-	+
HRPCa-2	8.5	4+4	4+5	cT1c	pT3bN1	+	+	+
HRPCa-3	4.6	4+3	3+4	cT2b	pT3aN0	-	+	+
HRPCa-4	36	4+3	4+4	cT2b	pT3bN1	+	+	+
HRPCa-5	6.07	4+5	3+4	cT2b	pT2cN0	-	-	-
HRPCa-6	2.09	4+5	3+4	cT2b	pT2bN0	-	-	-

^1^ seminal vesicle invasion.

^**2**^ surgical margin.

^**3**^ extracapsular extension.

### Precision-cutting and subrenal implantation of tissue slices

Male recombination activating gene-2 (RAG2)^−/−^γC^−/−^ mice bred at Stanford University or NIH III mice (Charles River, Wilmington, MA, USA) between 6 and 8 weeks of age were used. All animal studies were done in compliance with regulations at Stanford University. Precision-cutting and subrenal implantation of tissue slices were described previously [26]. A 25-mg testosterone pellet with a release rate of 0.2 mg/day was inserted into a small incision made under the skin between the shoulder blades.

### Castration of mice

Castration of mice was performed one month after subrenal implantation as previously described [26].

### Immunohistochemistry

Immunohistochemistry was performed as previously described [26]. Antigen retrieval was achieved by heating in citrate buffer (pH 6.0) for 20 minutes, followed by a 20-minute cool-down. The sources and dilutions of the antibodies used in this study are listed in Table 2.

**Table 2:** Antibodies used in the study

**Name**	**Source**	**Dilution**
Anti-cytokeratin 18 (K18)	Santa Cruz Biotechnology, Santa Cruz, CA	1:200
Anti-AR	BD Pharmingen, San Diego, CA	1:200
Anti-PSA	Santa Cruz Biotechnology	1:100
Anti-AMACR/p63	Biocare, Concord, CA	ready to use
Anti-Ki67	Biocare	1:100
Anti-Ku70	Abcam, Cambridge, MA	1:200
Anti-human specific-CD31	Dako Corp., Carpinteria, CA	1:20
Anti-CCNA	Leica Microsystems, Buffalo Grove, IL	1:50
Anti-ERG	Epitomics, Burlingame, CA	1:100
Anti-VIM	Epitomics	1:100
Anti-TOP2A	Dako Corp	1:50
universal biotinylated horse anti-mouse/rabbit IgG	Vector Laboratories Inc., Burlingame, CA	1:1000
Alexa 488 goat anti-mouse	Invitrogen, Carlsbad, CA	1:1000
Alexa 555 goat anti-rabbit	Invitrogen	1:1000

### Quantitation of Ki67 and CCNA expression

The proliferation index, defined as percentage of proliferating cells, was established by dividing the number of Ki67-positive cells by the total area of cancer (based on histology and/or AMACR expression) in ten 20X-microscopic fields, randomly chosen for each TSG. Similarly, the percentage of CCNA-positive cells was determined by dividing the number of CCNA-positive cells by the total area of cancer in five 20X-microscopic fields for each TSG. Student’s t-test with a significant level set at α < 0.05 was performed to determine statistical significance.

## Results

### Generation of TSGs from HRPCa

We generated 6–10 TSGs from each of 6 fresh HRPCa tissues obtained immediately following radical prostatectomy (Tables 1 and 3). Since high-grade cancer is likely the cause of recurrence after primary therapy, we excised tissues from areas containing mainly Gleason grade 4 and/or 5 cancer based on the ultrasound-guided prostate needle biopsy map obtained prior to surgery. While cutting at 300-μm, every other slice was frozen, sectioned, and stained with H&E to confirm histopathology. Only slices that were in-between two slices containing high-grade PCa were implanted in mice.

**Table 3:** Number of TSGs generated for each HRPCa case

	**Control TSGs**	**Castrated TSGs**	**Control TSGs containing cancer**	**Castrated TSGs containing cancer**
HRPCa-1	10^1^	NA	10	NA
HRPCa-2	5	3	5	3
HRPCa-3	3	3	3	3
HRPCa-4	5	5	5	3
HRPCa-5	4	5	4	0
HRPCa-6	4	4	4	0

^1^TSGs were harvested one month after implantation. All other TSGs were harvested two months after implantation.

**Figure 1:**
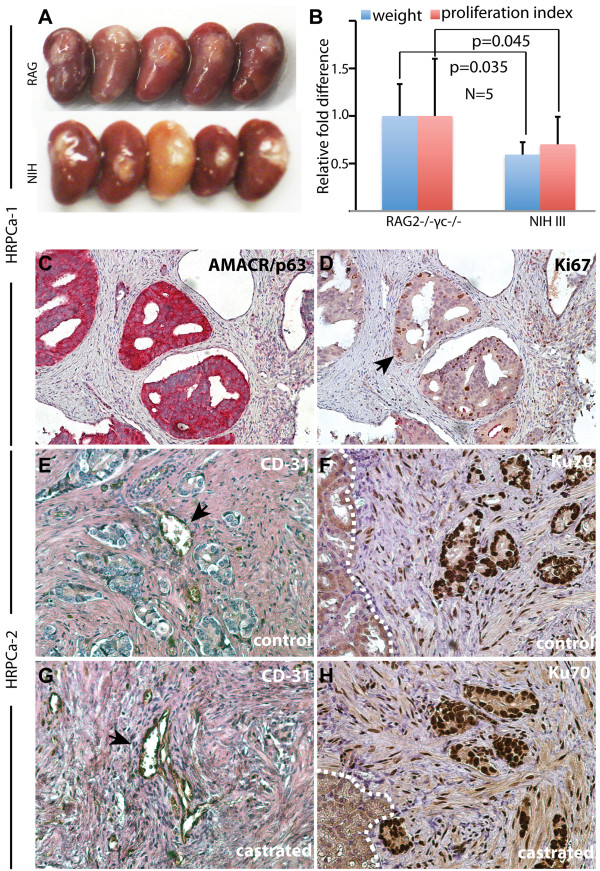
**Generation of TSGs from HRPCa-1 and −2. (A)** Kidneys with TSGs under the capsule harvested from RAG2^−/−^γc^−/−^ or NIH III mice; **(B)** both the graft weight and proliferation index of TSGs maintained in RAG2^−/−^γc^−/−^ mice were significantly higher than those maintained in NIH III mice by Student’s t-test; **(C**-**H)** immunohistochemistry of TSGs in RAG2^−/−^γc^−/−^ mice using antibodies against AMACR/p63 **(C)**, Ki67 **(D)**, human-specific CD31 **(E** and **G)**, and human-specific Ku70 **(F** and **H)**. **(E**-**F)** are control TSGs and **(G**-**H)** are TSGs one month after castration. Arrows in **(D)** and **(E** and **G)** point to Ki67- and CD31-positive cells, respectively. White dotted lines in **(F)** and **(H)** mark the boundary between mouse kidney and TSG. Magnification for **(C)** and **(D)** is 10X and **(E**-**H)** is 40X.

We first compared TSGs derived from the same parent tumor, HRPCa-1, but maintained in two mouse strains, RAG2^−/−^γc^−/−^[27,28] and NIH III [29,30]; both lack T, B, and natural killer cells but the extent of the deficiencies in each has not been established. Although the gross appearance of TSGs maintained in both hosts for 1 month was similar (Figure 1A), the average graft weight and the proliferation index of cancer in RAG2^−/−^γc^−/−^ mice were significantly higher than in NIH III mice (Figure 1B). Serial sections were stained with antibodies against AMACR to identify cancer cells and Ki67 to label proliferating cells (Figure 1C-D). The proliferation index in TSGs maintained in RAG2^−/−^γc^−/−^ mice was 97% of the parent tumor. These results suggest that RAG2^−/−^γc^−/−^ mice provide a more supportive environment for TSGs derived from HRPCa than NIH III mice and should be the host of choice for human PCa tumorgrafts.

Immunohistochemistry using human-specific CD31 antibody demonstrated that a considerable amount of the vasculature present in HRPCa TSGs two months following implantation was lined by endothelial cells of human origin, consistent with previous findings [25,26,31]. Representative images from HRPCa-2 are shown in Figure 1E. In addition, most of the stromal cells in TSGs are of human origin as demonstrated by labeling with an antibody against human-specific nuclear antigen Ku70 (Figure 1F). Similar results were observed in TSGs derived from the same parent tumor one month after castration (Figure 1G-H), demonstrating the persistence of human endothelial and stromal cells in prostate TSGs.

### TSGs derived from HRPCa resembled the parent tumors

**Figure 2:**
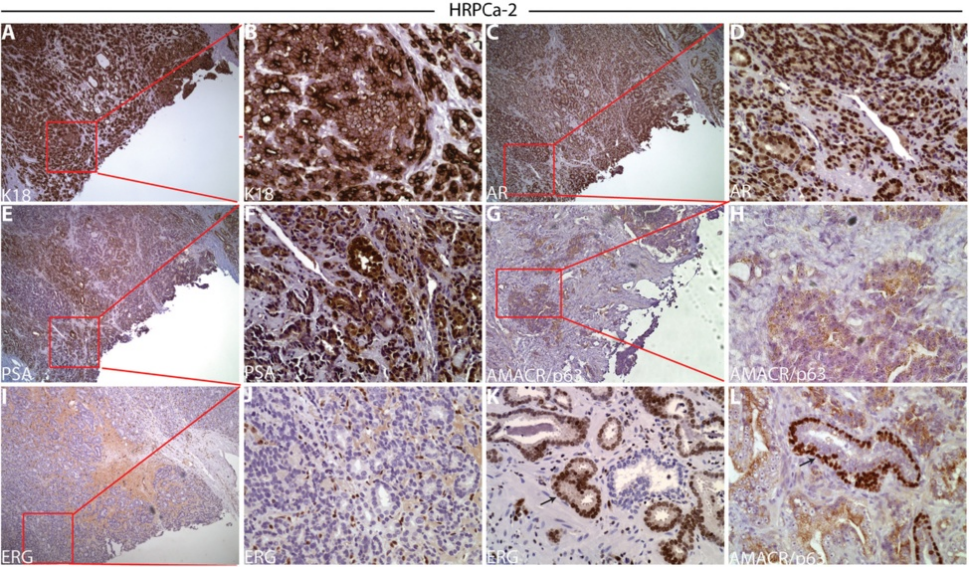
**Marker expression in HRPCa-2 by immunohistochemistry.** In the area where tissue cores were taken to derive TSGs, we observed: **(A**-**B)** strong cytoplasmic K18 staining; **(C**-**D)** nuclear staining of AR; **(E**-**F)** cytoplasmic staining of PSA; **(G**-**H)** negative staining for the normal basal cell marker p63; positive staining for cytoplasmic AMACR **(G**-**H)** and negative ERG **(I-J)**. In areas away from where tissue cores were taken in the same specimen, we observed nuclear ERG expression in low-grade cancer cells **(**arrows in **K)** and staining for nuclear p63 in normal basal epithelial cells **(**arrows in **L)**. **(B)**, **(D)**, **(F)**, **(H)**, and **(J)** are higher magnification (40X) of boxed areas in **(A)**, **(C)**, **(E)**, **(G)**, and **(I)** (10X), respectively. The ERG-positive cells in **(J)** are endothelial cells, not cancer cells.

We compared the histology and protein expression of cell type-specific markers in TSGs to the parent tumors. HRPCa-2, a parent tumor, expressed classic secretory cell markers including cytoplasmic K18 (Figure 2A-B), nuclear AR (Figure 2C-D), and cytoplasmic PSA (Figure 2E-F). In contrast, the tumor was negative for the basal epithelial cell marker p63 (Figure 2G-H). Moreover, the area where the tissue was taken for TSG generation was positive for AMACR (Figure 2G-H) but negative for ERG (Figure 2I-J), two markers widely used to identify PCa in humans. AMACR is expressed by ~90% of PCa [32] and is used to identify PCa in clinical specimens. Expression of ERG is highly correlated with the presence of the TMPRSS2-ERG gene fusion present in ~50% of PCa and is negatively correlated with Gleason score (i.e., high-grade PCa is less likely to express ERG) [33]. Consistent with the negative correlation between ERG expression and Gleason score, we observed ERG expression in grade 3 cancer in the same prostatectomy specimen (Figure 2K) as well as p63 staining in normal basal cells (Figure 2L), demonstrating that absence of staining in the area where the tissue was taken was not attributable to technical failure.

**Figure 3:**
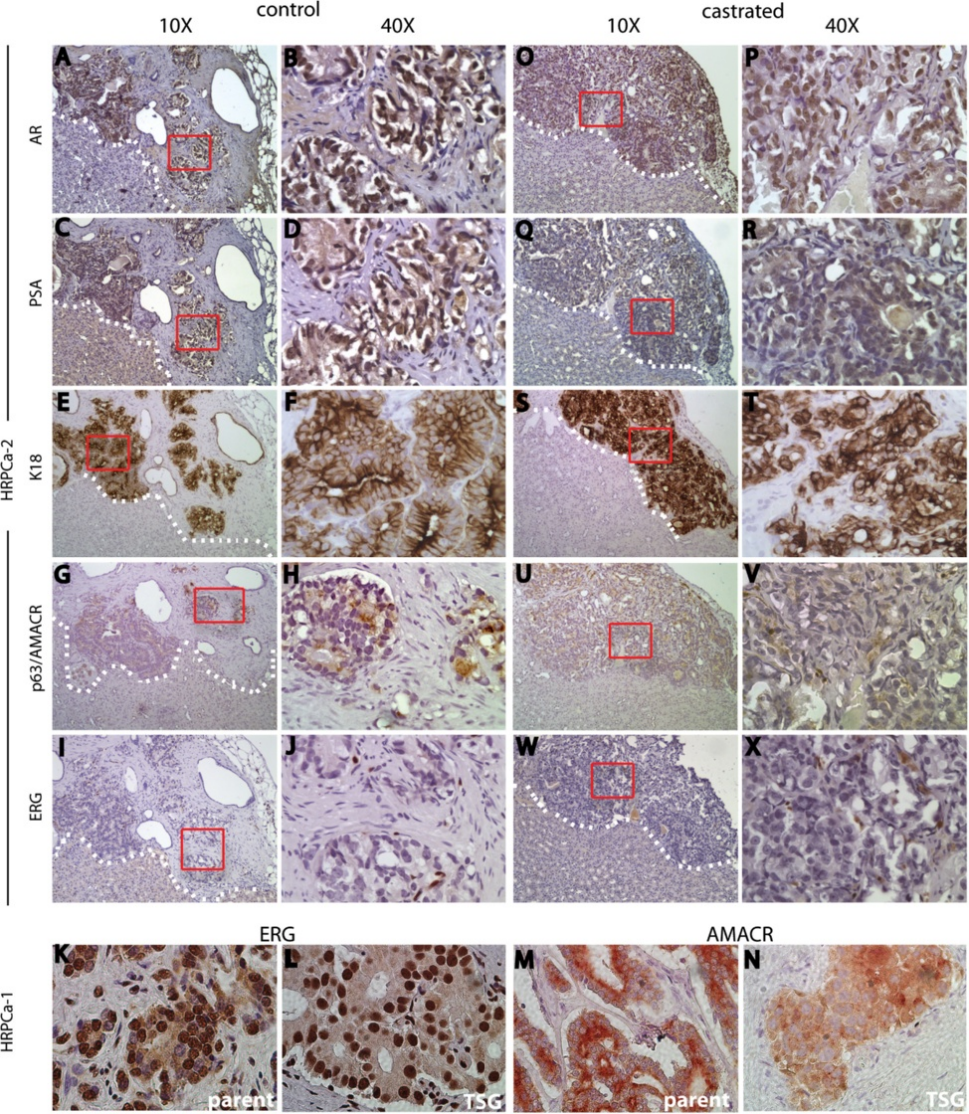
**Marker expression in TSGs derived from HRPCa-2.** In control TSGs derived from HRPCa-2, we observed: **(A**-**B)** nuclear staining of AR; **(C**-**D)** cytoplasmic staining of PSA; and **(E**-**F)** cytoplasmic staining of K18 in cancer cells. Positive staining of AMACR **(G**-**H)**, and negative staining of the basal epithelial cell marker p63 **(G**-**H)** and ERG **(I**-**J)** in cancer cells was also observed. ERG staining was present in endothelial cells **(I**-**J)**. In CR-TSGs derived from HRPCa-2, cancer cells showed moderate nuclear staining for AR **(O**-**P)**, little or no staining of PSA **(Q**-**R)**, and intense cytoplasmic staining for K18 **(S**-**T)**. Cancer cells were also positive for AMACR **(U**-**V)** and negative for ERG **(W**-**X)**. In HRPCa-1 and TSGs derived from it, we observed strong staining for ERG **(K**-**L)** and AMACR **(M**-**N)** in cancer cells. **(B)**, **(D)**, **(F)**, **(H)**, **(J)**, **(P)**, **(R)**, **(T)**, **(V)**, and **(X)** are higher magnifications of boxed areas in **(A)**, **(C)**, **(E)**, **(G)**, **(I)**, **(O)**, **(Q)**, **(S)**, **(U)** and **(W)**, respectively.

TSGs derived from HRPCa-2 harvested two months after implantation showed similar histomorphology to the parent tumor. Specifically, high-grade cancer was readily identifiable in these TSGs, appearing as an irregular mass of neoplastic cells with little or no gland formation (Figure 3). In addition, TSGs displayed similar expression of cell-type specific markers to the parent tumor (Figure 3A-J). TSGs from the other five HRPCa specimens also displayed similar histomorphology and marker expression as their parent tumors (Table 4). For example, both HRPCa-1 and its derived TSGs were strongly positive for ERG (Figure 3K-L) and AMACR (Figure 3M-N). Overall, these results demonstrated that high-grade cancer from HRPCa maintained appropriate histomorphology and protein expression in TSGs up to 2 months post-implantation.

**Table 4:** Marker expression in HRPCa where tissues were taken for TSG generation and in corresponding TSGs

**ID**	**K18**	**AR**	**PSA**	**AMACR**	**P63**	**ERG**
HRPCa-1	+	+	+	+	-	+
Derivative TSGs	+	+	+	+	-	+
HRPCa-2	+	+	+	+	-	-
Derivative TSGs	+	+	+	+	-	-
HRPCa-3	+	+	+	+	-	-
Derivative TSGs	+	+	+	+	-	-
HRPCa-4	+	+	+	+	-	-
Derivative TSGs	+	+	+	+	-	-
HRPCa-5	+	+	+	-	-	-
Derivative TSGs	+	+	+	-	-	-
HRPCa-6	+	+	+	+	-	-
Derivative TSGs	+	+	+	+	-	-

### Adverse pathological features of parent tumors predicted response of TSGs to ADT

**Figure 4:**
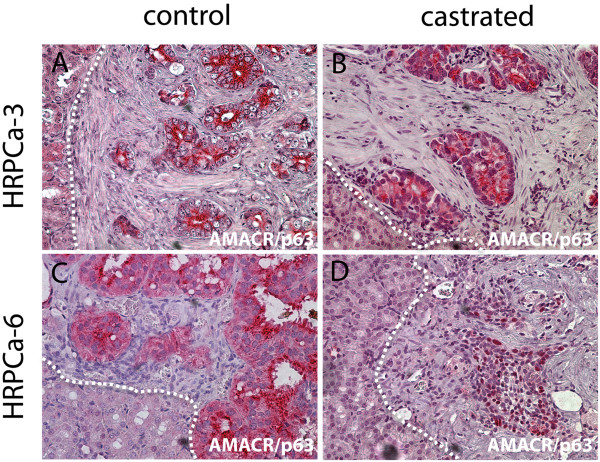
**Differential responses to ADT of TSGs derived from HRPCa with or without adverse pathological features.** Immunohistochemistry using an antibody cocktail for cytoplasmic AMACR and nuclear p63 demonstrated resistance of HRPCa-3, a HRPCa with adverse pathological features, to ADT **(A**-**B)**, and complete tumor regression in HRPCa-6, a HRPCa without adverse pathological features, after ADT **(C**-**D)**. In **(D)**, remnant epithelial cells are benign, as demonstrated by nuclear p63 staining. White dotted lines mark the boundary between mouse kidney and TSG. Magnification for all images is 40X.

Of the 6 cases used in this study, ADT was performed in 5 cases since HRPCa-1 was used for comparison of host mouse strains only. Two of the cases (HRPCa-5 and −6) were down-graded on final pathology of the radical prostatectomy specimens to Gleason score 7 from 9 and had no adverse pathological features such as positive surgical margin, seminal vesicle invasion, or extracapsular extension (Table 1). For accurate assessment effects of ADT, we assigned mice bearing TSGs derived from adjacent tissue slices into control and ADT groups. Interestingly, one month after ADT, cancer cells were found in 60-100% of the TSGs derived from HRPCa-2, -3, and −4 (Table 3). These TSGs were defined as castration-resistant TSGs (CR-TSGs). In contrast, no cancer cells were detected in TSGs derived from HRPCa-5 and −6, demonstrating complete tumor regression after ADT (Table 3). For example, TSGs derived from HRPCa-3 expressed a high level of AMACR in both control and castrated mice (Figure 4A-B), whereas AMACR-expressing cancer cells were only observed in TSGs derived from HRPCa-6 maintained in control but not castrated mice (Figure 4C-D). TSGs were sectioned throughout to confirm complete pathologic response to ADT (absence of AMACR-stained cells and no recognizable cancer by histopathological analysis). These results mimicked the heterogeneous response of HRPCa in patients to ADT, and suggest that adverse features of parent tumors may predict the response of TSGs to ADT.

### ADT modulated AR-regulated genes in TSGs similarly to PCa in humans

After ADT, remaining cancer cells in CR-TSGs derived from HRPCa-2 demonstrated similar histological features and biomarkers to those in control TSGs, including expression of AR (Figure 3O-P). As expected, these cells showed little or no staining for PSA (Figure 3Q-R), consistent with the response of PCa in humans to ADT [34]. Cancer cells were also positive for K18 (Figure 3S-T) and AMACR (Figure 3U-V), and negative for ERG (Figure 3W-X). These results suggest that ADT abolished PSA expression in cancer cells in CR-TSGs derived from HRPCa but did not affect expression of other markers.

**Figure 5:**
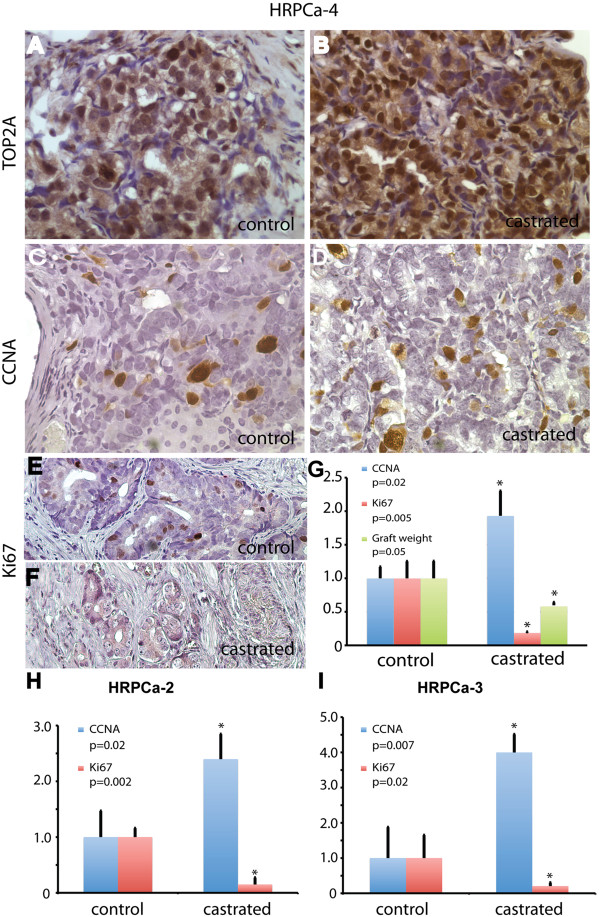
**Effects of ADT on AR target genes, cell proliferation, and graft weight in HRPCa-4 TSGs.** ADT dramatically increased the expression of TOP2A in CR-TSGs **(B)** compared to control **(A)**. Similarly, the number of CCNA-positive cells was 2.4-fold higher in CR-TSGs **(D)** that in control TSGs **(C)**. Ki67-positive cells were dramatically decreased in CR-TSGs **(F)** compared to control TSGs **(E)**. This decrease was statistically significant as was the decrease in graft weight in response to ADT by Student’s t-test **(G)**. ADT also decreased cell proliferation and upregulated CCNA expression in HRPCa-2 **(H)** and HRPCa-3 **(I)**. The values in castrated TSGs were normalized against control. * marks significant difference between castrated and control TSGs defined as p<0.05 by Student’s t-test.

We next examined the expression of AR-regulated genes that have been reported to be up-regulated in human CRPC including TOP2A and CCNA [35]. In CR-TSGs derived from HRPCa-4, the expression level and percentage of TOP2A-positive cells was significantly higher compared to control (Figure 5A-B). ADT also increased the percentage of CCNA-expressing cancer cells in CR-TSGs by 2.4-fold compared to control (Figure 5C-D, 5G). These results demonstrated that TSGs derived from HRPCa responded to ADT by upregulating AR target genes associated with CRPC in humans. Moreover, the number of Ki67-expressing cells in CR-TSGs was significantly lower than that in control (Figure 5E-G), consistent with the observation that ADT inhibits cell proliferation in PCa in humans [36]. Few or no apoptotic cells were detected in CR-TSGs by cleaved caspase-3 staining (data not shown); if apoptosis was induced by ADT, it may have occurred rapidly after castration and diminished by one month after castration. Similar results were observed for HRPCa-2 (Figure 5H) and −3 (Figure 5I). Together, these findings demonstrated an authentic response to ADT of HRPCa in TSGs similar to that occurs in humans, suggesting that CR-TSGs realistically model primary CRPC.

### ADT induced EMT in CR-TSGs similarly to human PCa

**Figure 6:**
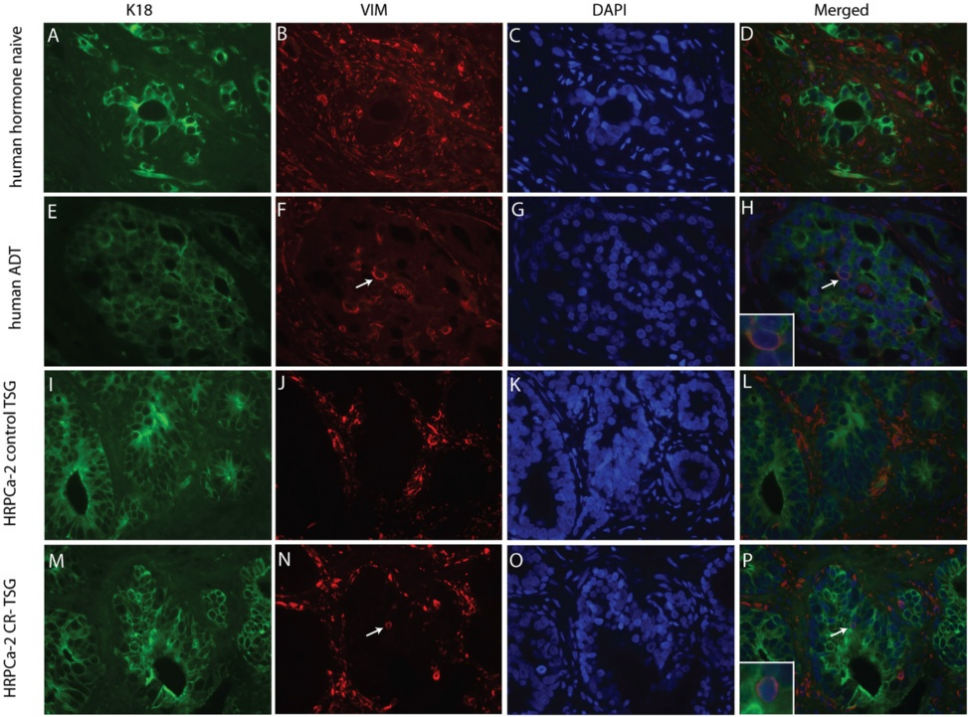
**ADT induced expression of VIM in PCa cells.** Double immunofluorescent staining showed that in human hormone-naive PCa, high-grade cancer cells showed strong cytoplasmic expression of K18 in green **(A)** and no expression of VIM **(B)**. VIM staining in red was present in the tissue (mostly in cytoplasm of stromal cells) but was exclusive from K18-positive cells **(B** and **D)**. In contrast, in human PCa treated with ADT, a small population of high-grade PCa cells expressed both K18 **(E)** and VIM **(**arrow in **F)** as shown in the merged image **(**arrow and insert in **H)**. In control TSGs derived from HRPCa-2, K18 **(I)** and VIM **(J)** displayed mutually exclusive staining **(L)**. In CR-TSGs, a rare population of cancer cells expressing both VIM **(**arrow in **N)** and K18 **(M)** was observed in the merged image **(**arrow and insert in **P)**. **(C)**, **(G)**, **(K)**, and **(O)** showed nuclear DAPI staining of the same cells in **(A**-**B)**, **(E**-**F)**, **(I**-**J)**, and **(M**-**N)**, respectively. Magnification for all images is 40X.

To determine the effects of ADT on EMT in TSGs and compare to its effects in PCa in humans, we examined EMT marker expression in three human prostates removed by radical prostatectomy following neoadjuvant ADT (Flutamide or Lupron treatment of 6 to 10 weeks in duration) and two untreated specimens as controls. In untreated PCa, high-grade cancers of Gleason patterns 4 and 5 showed strong cytoplasmic expression of K18 (Figure 6A), a classic epithelial cell marker. Expression of VIM, a mesenchymal marker whose expression is increased in epithelial cells during EMT, was only observed in stromal cells (Figure 6B) and mutually exclusive from K18 expression (Figure 6D). In contrast, a small population of high-grade PCa cells expressed both K18 (Figure 6E) and VIM (arrows in Figure 6F and 6H) in specimens treated with ADT. Although VIM-positive, these cells still maintained epithelial cell morphology, i.e., cuboidal rather than spindle-shaped. These results suggest that a subset of high-grade prostate cancer cells in humans treated with ADT underwent EMT.

**Figure 7:**
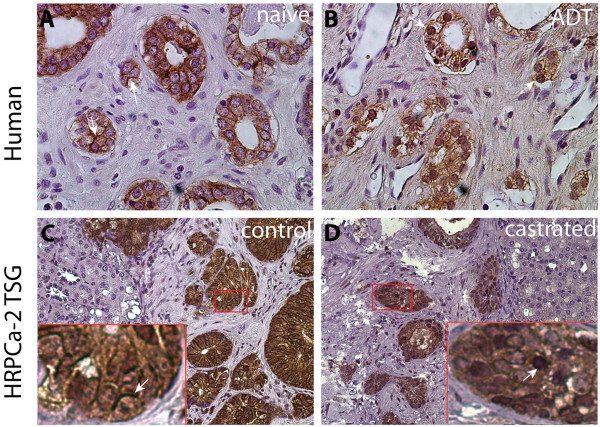
**ADT induced mislocalization of E-cadherin in TSGs derived from HRPCa-2.** E-cadherin, a well known epithelial marker, was primarily localized on cell membranes in cancer cells of human hormone-naive PCa **(A)** and control TSGs derived from HRPCa-2 **(C)**, while in human PCa treated with ADT **(B)** and castrated TSGs **(D)**, it was mislocalized away from the cell membrane **(**arrow in **C)** into the nucleus **(**arrow in **D)**. The inserts are enlarged images of the boxed area in each image. Magnification for **(A)** and **(B)** is 40X, and for **(C)** and **(D)**, 20X.

A similar staining pattern was observed in CR-TSGs derived from HRPCa-2, -3 and −4. Specifically, a rare population of cancer cells expressing both VIM and K18 was observed in CR-TSGs (Figure 6M-P) but not in control TSGs (Figure 6I-L). Moreover, E-cadherin, a well-known epithelial marker, was primarily localized on the cell membrane in cancer cells of control TSGs, while in CR-TSGs, it was mislocalized away from the cell membrane into the nucleus (Figure 7). This leads to loss of function of E-cadherin, commonly observed during EMT [37,38]. These results suggest that ADT induced EMT in the TSG model similar to CRPC in humans.

## Discussion

We have shown that TSGs derived from HRPCa recapitulate characteristics of parent tumors including histopathological features, biomarker expression, and responses to ADT. Our study differs from previous reports [21-26] of generation of prostate tumorgrafts in several ways. First, we implanted precision-cut tissue slices rather than minced tissues as used in previous studies. The ability to reliably determine the presence and grade of cancer prior to implantation is one of the advantages of this methodology, which in turn allows more accurate assessment of therapeutic effects in large-scale animal trials. For example, our study design ruled out the possibility that “bad” tumors were missed in the two cases that showed complete regression after castration. Because we used only slices that were in-between two slices containing high-grade PCa and assigned adjacent tissue slices to control and ADT groups, it is unlikely that the “bad” tumors were missed only in the ADT but not the control group. If minced tissues were used without prior knowledge of histopathology, it would be impossible to know whether the complete regression was just by chance, i.e., the bad tumors were missed or there was no cancer present initially in the ADT group. Second, we focused on high-risk tumors, the major contributor to PCa mortality, rather than benign or low-risk tumors that have been used in most previous studies. Third, we systematically evaluated the effects of ADT, a standard treatment for HRPCa, in our TSGs. Although the number of cases in our study is small, it is the largest cohort of HRPCa evaluated for response to ADT to date in a preclinical model.

HRPCa is the target of adjuvant and neoadjuvant therapies since low-risk PCa is largely curable by surgery or radiation or needs no treatment. A growing inventory of new agents has been discovered that may improve the clinical outcome of HRPCa. Clinical trials evaluating such candidate compounds require a large number of patients, are expensive and time-consuming, and expose patients to certain risks. The TSG model of HRPCa provides a much-needed pre-clinical screening platform that can be used to rapidly narrow down the number of agents or regimens for further investigation in clinical trials. The authenticity of the model in recapitulating the features of the parent tumors increases confidence in the likelihood of similar drug responses in humans. In addition, our study demonstrates the feasibility of generating a relatively large number of TSGs from the same HRPCa specimen with similar cell composition and histology among control and experimental samples in an *in vivo* setting. This capability is particularly useful since PCa specimens are becoming smaller due to early cancer detection. Our model can be used to test a variety of therapeutic strategies, including potential curative therapies for HRPCa that can either prevent CRPC from arising during ADT or kill CRPC cells after disease progression. Since ADT may be associated with numerous side effects such as increased cardiovascular mortality, other alternative therapies should also be investigated [39]. Finally, our model can be used to better understand the mechanisms of development of CRPC, which will in turn accelerate the discovery of effective therapies.

As proof-of-principle, we have demonstrated that our model closely mimics the response of PCa in humans to ADT. First, ADT decreased cell proliferation and reduced graft weight of TSGs. Second, ADT downregulated the conventional AR target gene PSA while selectively upregulating CCNA and TOP2A in CR-TSGs, as in human PCa [35], suggesting that the TSG model is a suitable platform for pre-clinical testing of the ever-growing number of new therapeutic agents that aim to better prevent AR activation in CRPC. Third, consistent with recent studies highlighting a role for EMT after ADT in facilitating human PCa progression and metastasis [14,40,41], cancer cells in CR-TSGs exhibited EMT by simultaneously expressing both mesenchymal and epithelial cell markers, VIM and K18, respectively. In addition, E-cadherin was mislocalized away from cell membranes into the nuclei in CR-TSGs, presumably disrupting the function of E-cadherin in preventing beta-catenin from entering the nucleus [38]. Such mislocalization was recently observed in a metastatic colorectal cancer model in which E-cadherin nuclear translocation was associated with aggressive focal growth [42], suggesting that mislocalization of E-cadherin may be a general mechanism of cancer progression. The documentation of ADT-induced EMT in CR-TSGs derived from HRPCa suggests an attractive model for testing novel therapeutics aimed at blocking EMT.

Our findings are the first to link seminal vesicle invasion, positive surgical margin and extracapsular extension to lack of complete pathologic response to ADT by HRPCa. The efficacy of neoadjuvant ADT in the TSG model appears much better than in patients determined by histology [43,44]. Since the presence or absence of tumor cells in TSGs was evaluated one month after castration, we can’t rule out the possibility that the regressed tumors might relapse at later time points. In addition, most studies show a lower serum testosterone level in castrated mice than in humans [26,45-47], possibly because unlike in humans, adrenal glands in mice do not produce androgen [48-50]. Thus, castration of mice may more effectively eliminate HRPCa cells in TSGs than does ADT in humans. Further experiments are needed to determine the long-term effects of ADT and to investigate the possibility of serial passage in this model. Mechanisms of resistance to therapy can be explored, such as the role of stem cells in castration-resistance.

It is interesting to note that endothelial and stromal cells in TSGs are mostly of human origin, rather than replaced by their host counterparts. This is consistent with a recent report demonstrating a burst of angiogenesis by endogenous human blood vessels in primary xenografts of benign prostate or PCa tissues that occurred between days 6–14 after transplantation into SCID mice pre-implanted with testosterone pellets [25]. In contrast, DeRose et al. demonstrated that, in human breast tumorgrafts, cancer-associated stroma and endothelial cells from the original tumor were largely replaced by mouse-derived stroma and endothelial cells [51]. This difference may be due to the disparate growth properties of these two types of cancers − PCa is a slow-growing cancer with a long natural history, whereas breast cancer is much more aggressive. The slow-growing nature of prostate TSGs perhaps makes it unnecessary to incorporate host stromal and endothelial cells in the grafts. In our study, human stromal and endothelial cells survived up to 2 months in TSGs derived from HRPCa. It would be interesting to determine whether the human endothelial and stromal cells would eventually be replaced by their mouse counterparts in long-term follow-up of these grafts.

## Conclusions

We provide evidence that TSGs are a realistic model of primary HRPCa and CRPC that may be used with high predictive power to evaluate the exponentially growing number of molecularly targeted therapies and to discriminate the most effective therapeutics for further clinical development.

## Abbreviations

ADT: Androgen deprivation therapy; AR: Androgen receptor; CRPC: Castration-resistant prostate cancer; CR-TSGs: Castration-resistant tissue slice grafts; EMT: Epithelial-mesenchymal transition; PCa: Prostate cancer; PSA: Prostate-specific antigen; TSG: Tissue slice graft.

## Competing interests

The authors declare that they have no competing interests.

## Authors’ contributions

HZ conceived of the study, participated in its design, carried out the TSG generation and castration, analyzed data and drafted the manuscript. AT and AI assisted with TSG generation and castration. RN, SWR, and JS carried out the immunostaining. DMP conceived of the study, and participated in its design and revision of the manuscript. All authors read and approved the final manuscript.
